# Plutonium isotopes in the North Western Pacific sediments coupled with radiocarbon in corals recording precise timing of the Anthropocene

**DOI:** 10.1038/s41598-022-14179-w

**Published:** 2022-07-01

**Authors:** Yusuke Yokoyama, Stephen Tims, Michaela Froehlich, Shoko Hirabayashi, Takahiro Aze, L. Keith Fifield, Dominik Koll, Yosuke Miyairi, Stefan Pavetich, Michinobu Kuwae

**Affiliations:** 1 Atmosphere and Ocean Research Institute, The University of Tokyo, 5-1-5 Kashiwanoha, Kashiwa, Chiba 277-8564 Japan; 2 Department of Earth and Planetary Sciences, Graduate School of Science, The University of Tokyo, 7-3-1 Hongo, Bunkyo-ku, Tokyo, 113-8033 Japan; 3 Graduate Program on Environmental Sciences, The University of Tokyo, 3-8-1 Komaba, Meguro-ku, Tokyo, 153-8902 Japan; 4 Biogeochemistry Research Center, Research Institute for Marine Resources Utilization, Japan Agency for Marine-Earth Science and Technology, 2-15 Natsushima-cho, Yokosuka, Kanagawa 237-0061 Japan; 5 Research School of Physics, The Australian National University, Canberra, ACT 2601 Australia; 6 Center for Marine Environmental Studies, Ehime University, 2-5 Bunkyo-cho, Matsuyama, Ehime 790-8577 Japan

**Keywords:** Sedimentology, Geochemistry

## Abstract

Plutonium (Pu) has been used as a mid-twentieth century time-marker in various geological archives as a result of atmospheric nuclear tests mainly conducted in 1950s. Advancement of analytical techniques allows us to measure ^239^Pu and ^240^Pu more accurately and can thereby reconstruct the Pacific Pu signal that originated from the former Pacific Proving Grounds (PPG) in the Marshall Islands. Here, we propose a novel method that couples annual banded reef building corals and nearshore anoxic marine sediments to provide a marker to precisely determine the start of the nuclear era which is known as a part of the Anthropocene. We demonstrate the efficacy of the methods using sediment obtained from Beppu Bay, Japan, and a coral from Ishigaki Island, Japan. The sedimentary records show a clear Pu increase from 1950, peaking during the 1960s, and then showing a sharp decline during the 1970s. However, a constantly higher isotope ratio between ^239^Pu and ^240^Pu suggest an additional contribution other than global fallout via ocean currents. Furthermore, single elevations in ^240^Pu/^239^Pu provide supportive evidence of close-in-fallout similar to previous studies. Coral skeletal radiocarbon displays a clear timing with the signatures supporting the reliability of the Beppu Bay sediments as archives and demonstrates the strength of this method to capture potential Anthropocene signatures.

## Introduction

The Earth and environmental sciences allow us to understand changes in the planets’ status through time. Determination of the boundary of geological epochs that represent major shifts in the Earth system and have therefore received interest from a wide range of research fields. Great efforts have been made by the international scientific community to define official geological boundaries associated with major global environmental events, often related to changing interactions amongst Earth’s sub-systems (e.g., Refs.^1–6^). The present day geological era is the Holocene epoch which started from 11,700 years ago (11.7 ka), and is further divided into the Greenlandian Age (11.7–8.2 ka), Northgrippian Age (8.2–4.2 ka) and Meghalayan Age (4.2 ka-present) according to the ICS (International Commission on Stratigraphy) of the IUGS (International Union of Geological Sciences). However, since the mid twentieth century, industrialization, energy usage, and the global population and economy have increased dramatically, severely impacting Earth’s environment in what is often referred to as the great acceleration^7^. Because of this, the AWG (Anthropocene Working Group) of the ICS is now working towards defining the time that marks the end of the Holocene and the start of the Anthropocene^8^. The majority of the AWG group favor the use of plutonium isotope signals recorded in geological samples as a stratigraphic marker because values sharply increased during the 1950s and became widely distributed due to above ground thermonuclear bomb tests^9,10^. At present however, the use of plutonium signals as a stratigraphic marker for the start of the Anthropocene has only limited acceptance, and there is no consensus on a date that delineates the boundary between the Holocene and the Anthropocene. In what follows we have used the term Anthropocene to refer to the time period commencing with the appearance of plutonium signals in the geological record that are attributable to the first atmospheric nuclear weapons tests, namely in the period from 1954 (CASTLE BRAVO test, see below) to the present.

**Figure 1:**
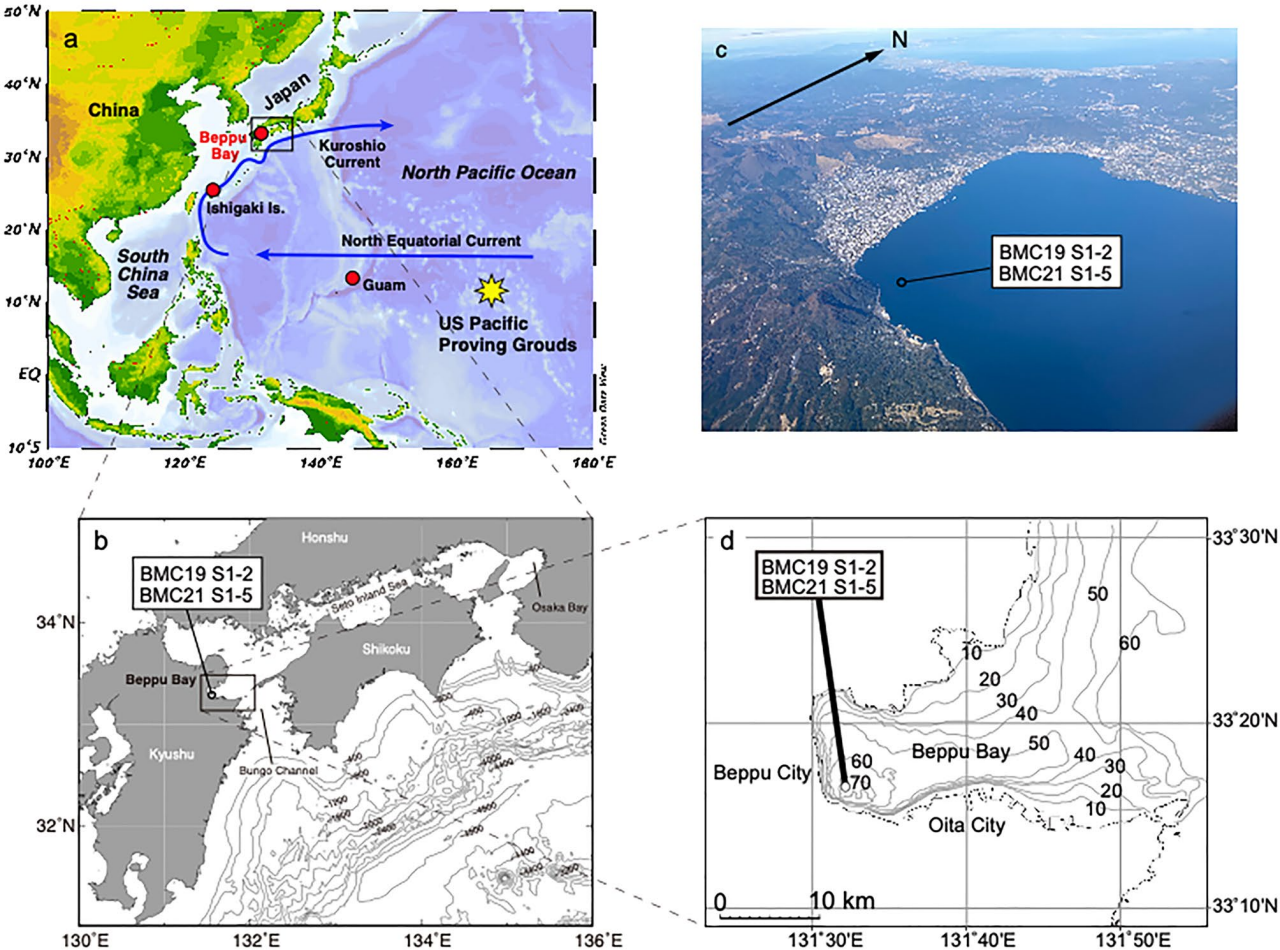
Location of Beppu Bay and key locations in the North Pacific Ocean. (**a**) The US pacific proving ground (PPG) is located east of Guam, and the North Equatorial Current (NEC) and Kuroshio Current (KC). The software Ocean Data View Ocean Data View 5.4.0 (Schlitzer, R., Ocean Data View, http://www.awi-bremerhaven.de/GEO/ODV,2003.) was used to draw the map. (**b**) Beppu bay is located at the entrance of Setonaikai Inland Sea and is connected to the Pacific Ocean via the Bungo Channel. Map was generated using GMT with topographic data of ETOPO1 (https://www.ngdc.noaa.gov/mgg/global/global.html). (**c**) Aerial photo of Beppu Bay. The coring site for this study is also indicated. (**d**) Contours indicating the water depth in meters showing anoxic bottom water conditions are created by the depicted bathymetric feature. The map was drawn using the chart distributed by the Hydrographic and Oceanographic Department, Japan Coast Guard (https://www1.kaiho.mlit.go.jp/jhd-E.html).

Prior to 1963 CE (Common Era), when the Partial Test Ban Treaty (PTBT) was issued to limit above ground nuclear bomb tests, approximately 3 tons of ^239^Pu had been released to the environment^9^. Global fallout of Pu that resulted from the tests, which commenced in 1945 CE, peaked in the Northern Hemisphere between 1961 and 1962 CE. Various geological archives have been considered as the best recorder of the Anthropocene^11^. One of these is reef building corals since their annual banded calcium carbonate skeleton can record signals of ambient seawater^12–15^. Age models are often reconstructed using these growth bands determined from X-ray images. However, miscounting of bandings are reported in several occasions that sometimes produce confusing pictures of the timing of Pu signals. For example, a coral obtained from Ishigaki Is. in Japan (Fig. 1a), located downstream of the Kuroshio Current (KC), displayed an earlier arrival of Pu signals than those collect from Guam^16^ (Fig. 1a). Accordingly, a thorough examination is required to establish the age model, employing a variety of independent layer counting methods. Clearly, corals can record climate signals such as temperature and salinity to understand the changes before and after the Anthropocene. Long-term baseline assessments (ca. millennial or longer) of environments are limited though, since the time span of typical long-lived corals only cover less than 100 years^17,18^, which is far shorter than the duration of the Holocene epoch (11.7 ka).

High accumulation of sediment at coastal marine sites, in particular the anoxic ocean floor, has an advantage from this perspective since records can extend back further to cover the entire Holocene^19^. Marine environmental information is retained within various sediment proxy records, e.g. oxygen isotopes, trace element geochemistry, organic geochemistry and so forth^20–23^. Therefore, sediments, in particular obtained from coastal sites are well suited to discuss local environmental changes closely related to anthropogenic activities in the context of global environmental changes^24^.

Plutonium in marine sediments has been measured from the open ocean to coastal areas^25,26^ for the last several decades, including at several sites across the Pacific Ocean^27^ where numerous nuclear weapons were tested. Thus, studies concerning airborne global fallout and fallout transported by ocean currents (and derived from close-in fallout at the test sites) exist^28^. Because the different nuclear test series created a variety of ^240^Pu/^239^Pu signatures, the source of the Pu can be traced^25^. The average ^240^Pu/^239^Pu ratio of ca. 0.18 is reported for global fallout^29,30^. For the case of the North Pacific region, the US Pacific Proving Ground (PPG) in the Marshall Islands was used as a test site during the 1950s to 1960s, and corals and soil samples showed elevated ratios as high as 0.30–0.35^31–33^. This was attributed largely to the CASTLE BRAVO test which took place in March 1954^33^.

The North Pacific Ocean is characterized as a location downstream of the North Equatorial Current (NEC) and Kuroshio Current (KC), which have average current speeds of 0.1–0.2 m s^−1^ and 0.5–1 m s^−1^, respectively. The wide distribution of Pu in the region shows that particulates originated from the PPG and were then transported by the NEC and KC. Compilation of previously reported Pu isotope studies in the region show that a ^240^Pu/^239^Pu higher than ca. 0.2 is found in sediments and corals as well as seawater in widespread regions of the North Pacific^26^. A high atomic ratio of Pu isotopes (^240^Pu/^239^Pu) of more than ca. 0.3 is known as observed in PPG material^32^. This is significantly larger than that at all other known nuclear test sites. Hence ^240^Pu/^239^Pu atomic ratios higher than 0.2 are attributed to the influence of materials transported from the Marshall Islands. This is in line with seawater ^137^Cs measurements conducted in the 1950s^34^. An isotopic mass balance model^35^ indicates 30–40% of the Pu in the North Pacific derives from the PPG via the NEC/KC system, including in the years after 1963 when the PTBT (Partial Test Ban Treaty) came into effect. Sediment trap and seawater samples collected along the KC in southern Japan show higher Pu isotopic ratios (> 0.18), and hence lateral movement of PPG sourced dissolved Pu^35^. Other studies investigating the NEC-KC pathways confirm this observation^27,28,35–42^. Thus, detecting the arrival signature of PPG derived Pu, as characterized by high Pu-isotope ratios, can assign the timing in the material as recently following the CASTLE BRAVO test of March 1954. In sum, the North Western Pacific Ocean basin has a great advantage since reef building coral and PPG signals can be used to identify the timing of anthropogenic signatures namely nuclear bomb tests.

Here, we propose a novel method that couples annual banded reef building corals and nearshore anoxic marine sediments with the former age determinations being made by X-ray photos and a high-resolution sea surface temperature proxy (Sr/Ca) to precisely determine the start of the Anthropocene using global fallout signals. We demonstrate the efficacy of the methods using sediment obtained from Beppu Bay, Japan, and a coral from Ishigaki Island, Japan (Fig. 1).

### Locations and samples

The Japanese archipelago is located in the North West Pacific where the warm KC flows offshore of the southern half of the islands (Fig. 1). The KC originates near the North-Eastern parts of the Philippines where the North Equatorial Current (NEC) bifurcates in meridional directions. Sediment cores obtained for this study were retrieved from the deepest part of the Beppu Bay, east of Kyushu (33° 16′ 23.5″ N, 131° 32′ 21.5″ E, 70 m water depth), where anoxic bottom water conditions persist from boreal spring to fall due to a shallow sill (ca. 50 m water depth) at the mouth of the bay (Fig. 1). Well preserved past oceanic and environmental conditions are expected, as confirmed by previous studies^19,43,44^.

**Figure 2:**
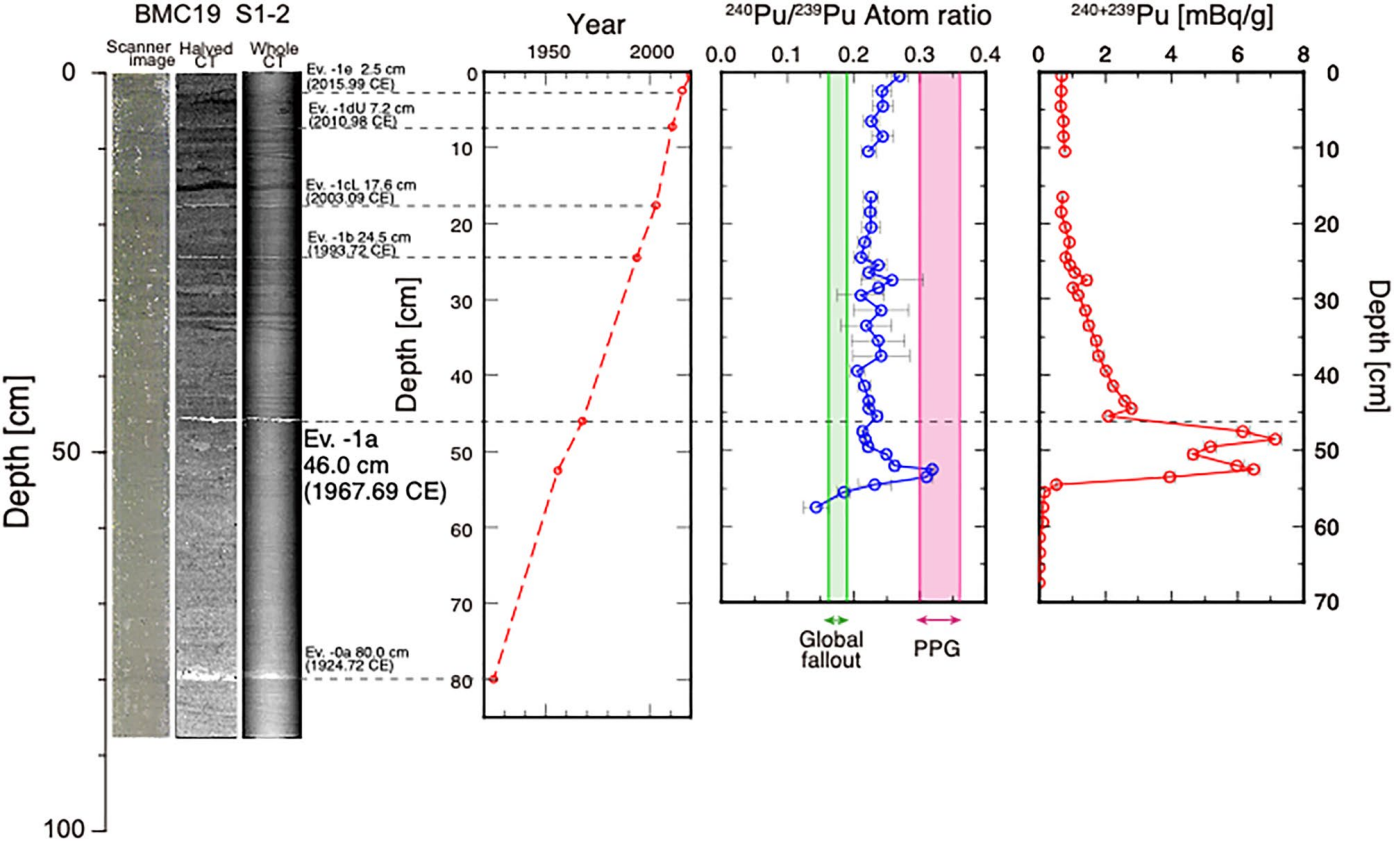
CT scan image of the Beppu Bay sediments and Pu measurements using Accelerator Mass Spectrometer (AMS). Horizons of geological event layers (e.g., Ev-1e, Ev-1dU etc.) can be recognized in images of cores and are traceable amongst cores taken from the basin^24^. They are formed by turbidite created from Tsunamis and other events. Ev.-1a at 46.0 cm bsf is recorded in historical documents with an age consistent with the Pb-210 age model. Pu isotope ratios and total activities are measured using Accelerator Mass Spectrometry^63^.

The cores were collected using a 120 cm long multiple corer, carefully preserving the sediment water interface^1,43,45^. They underwent sedimentological analyses including CT-scanning. Sediments consist of a silty clay with the occasional occurrence of distinct centimeter thick coarse layers, visible in CT-scan image (Fig. 2). They are likely formed due to geological events such as flooding and earthquakes^1^. The layers are readily traceable between the two cores and useful to correla te with each other (Fig. 2 & Fig. S1).

## Results

Radioactivity was measured to identify the horizon of the global fall out maximum in the BMC19 S1-3 core (Supplementary Table S1). It is known that the signal in Japan is widely observed a year later, according to monthly measurements for atmospheric deposition samples since 1956^46^. For example, observations of ^137^Cs in Japan display a 1963 CE peak associated with many nuclear bomb tests conducted in 1961 and 1962 CE. The constant rate of supply (CRS) age/depth model using Pb-210^47^ was also applied to the sediment core^43^. Forty samples were measured for Pu-isotopes using AMS on the BMC19 S1-2 core and ^239+240^Pu activities ranged from 0.6 to 6.5 mBq/g for the upper 54 cm, whereas almost no Pu activities were detected below the 54 cm bsf (below seafloor) horizon (Supplementary Table S1). A Pu isotopic ratio higher than 0.17 was observed for most of the cores above the 55 cm bsf horizon. Pu activities correspond to background levels for the AMS technique below 55 cm bsf, and hence analytical uncertainties associated with the isotope ratios for these samples are exceptionally large. Twelve samples of Plutonium isotope measurements were conducted on another core, BMC21S1-5 (Supplementary Table S2).

## Discussion

**Figure 3:**
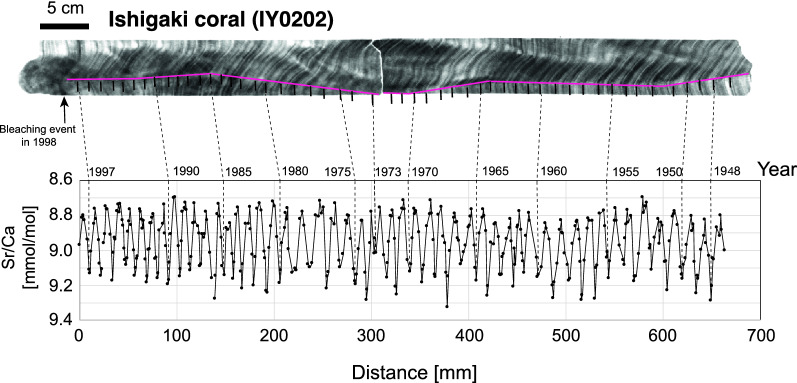
Coral X-ray photos and corresponding Sr/Ca for coral obtained from Ishigaki Is. Age model of the corals discussed here (i.e. from Ishigaki^13^ and from Guam^57^) are established through coupling layer counting of X-ray images and Sr/Ca measurements of corals. Sr/Ca can provide sea surface temperature information so that growth bands of corals can independently warranted.

Rapid growth (> 1 cm/year) of reef building corals can record high resolution seawater information in their calcium carbonate skeleton (Fig. 3). Seasonal growth patterns can be traced by X-ray images, which can be counted like tree rings, and isotopes, while trace elements in the skeleton can be used to monitor various oceanographic conditions^48^. Counting these seasonal density bands can provide reliable age information. However, the density bandings are sometimes unrecognizable which can cause the problem of miscounting the layers. This difference can be as much as 5–7 years^49,50^, creating a significant issue for high resolution oceanographic record preserved in coral skeletons. Therefore, an independent chronology to validate the X-ray image age model is necessary. Skeletal Sr/Ca is one possibility as the records provide sea surface temperature reconstructions since Sr contents in the skeleton are inversely correlated with SST^51,52^. Thus, the present study established the age model using combined X-ray and Sr/Ca measurements applying newly developed accurate measurements^53,54^ to warrant the age model (Fig. 3, Supplementary Table S3).

**Figure 4:**
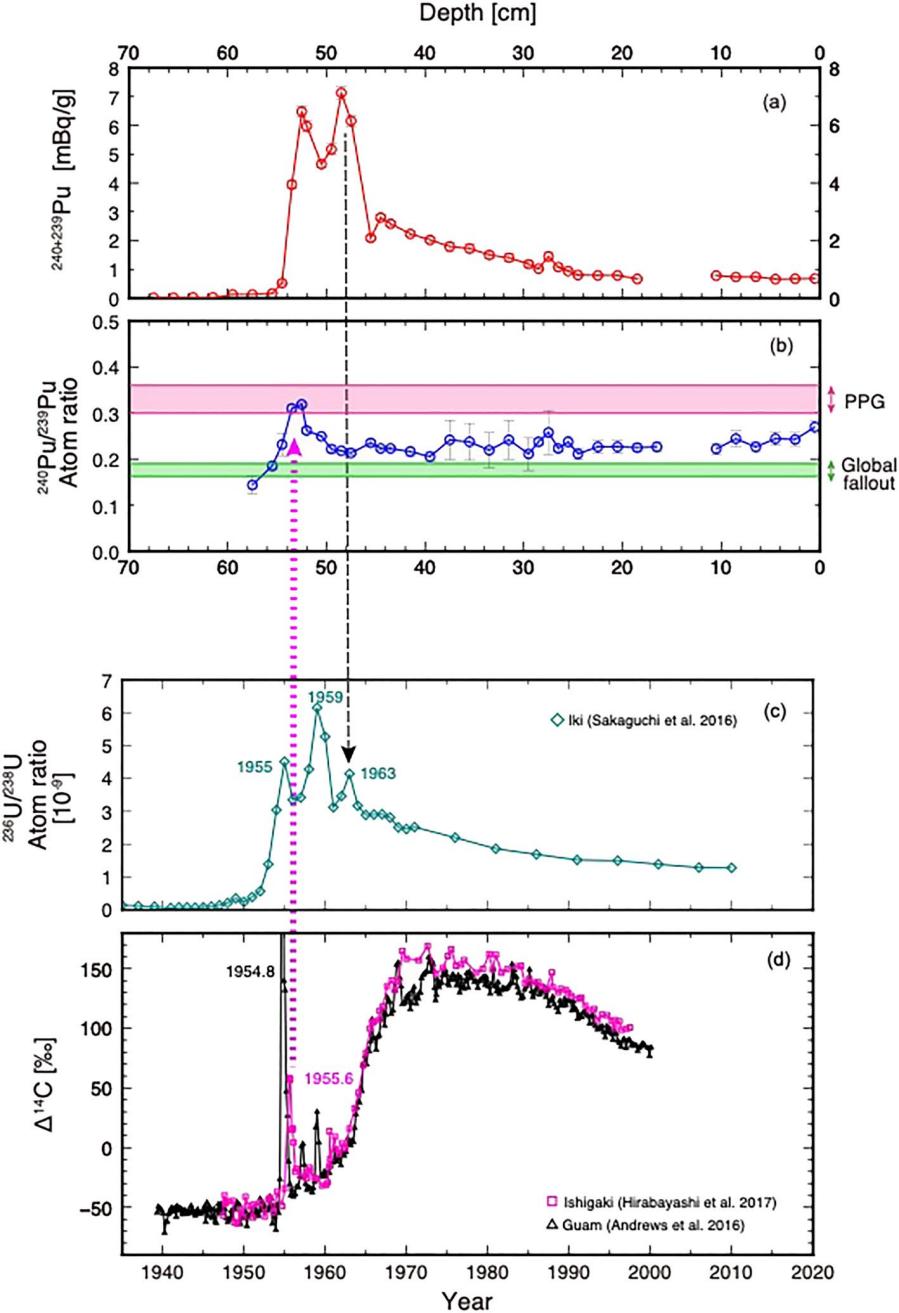
PPG and the Global fallout recorded in the Beppu Bay sediments and coral radiocarbon and uranium measurements. Age model of the sediment taken from Beppu Bay is reconstructed using radioisotopes measurements in corals from Japan. A sharp Δ^14^C peak found in Ishigaki Coral at 1954.8 CE was attributed as the close in fallout signature^13^ which is consistent with uranium isotope ratio data obtained from Iki Is^59^. Since Pu isotopes in Beppu Bay sediments clearly show a PPG value at 52.5 cm bsf in the core BMC19, it can be assigned to 1955.6 CE (Red dotted arrows). A linear sedimentation rate is applied to the section between this depth and 24.5 cm bsf (Ev.-1b; 1993.72 CE). The drop in total Pu was attributed to 1963 CE which is consistent with the uranium peak found in corals obtained from Iki (black dotted line), supporting the validity of the Beppu Bay sediment age model. The stratigraphy also verifies that the age of Ev.-1a, found at 46.0 cm bsf as derived from the age model as 1968 CE, is consistent with historical documents of the Hyuganada Earthquake Tsunami.

Various studies have used radiocarbon as a tracer to monitor the re-distribution of above ground nuclear bomb test fallout. Conventionally, the rise in radiocarbon content that occurred during the 1960s is used to monitor the transfer of radiocarbon from the atmosphere to the surface ocean^55,56^. However recent high-resolution measurements (i.e. seasonal to bi-monthly) on coral skeletons have shown that close-in fallout signatures can be traced using radiocarbon for some of the high fission tests conducted in 1950s^13,14,57,58^. An almost sixfold high short-lived radiocarbon peak is recorded in the coral skeleton obtained from Guam, the timing of which was inferred by the coral to be 1953 CE^57^ (Fig. 4d). The peak was interpreted as the close-in fallout materials transported westward from the PPG by ocean currents (i.e. NEC). This signal is also found in coral from Ishigaki, southern Ryukyu Japan, situated downstream of the KC, with an increase in radiocarbon by as much as 219 permil at 1955.6 CE^13^ (Fig. 4d). This indicates an average NEC-KC transport speed of 0.2 m s^−1^ which is in agreement with the value derived from uranium measured using coral from Japan^59^ (Fig. 4c), and provides strong support for the identification of the signal as attributable to the series of large tests carried out at the PPG in March and April 1954, which included the 15 MT (TNT equivalent) CASTLE BRAVO test (Supplementary Table S3). In addition, the coral record results from Ishigaki Is., Japan are supported by uranium isotope signals from Iki Is., Japan (Fig. 4c,d) and suggest that the arrival of the PPG signal at the Japanese water was 1955.6 CE^13^.

Plutonium isotopes are a unique proxy for understanding the source of nuclides^32^, and several studies using sediments from the North Pacific have been reported. One such site is the sediments from Sagami-bay off Tokyo which was investigated for downcore variation in Pu-isotopes^37^. The highest broad peak of ^240^Pu/^239^Pu (ca. 0.27) did not appear in the same horizon as the ^137^Cs peak from the same sediment core but was found in a deeper layer. The authors concluded that the ^240^Pu/^239^Pu peak was due to the 1950s PPG close-in fall out signal, whereas the peak in both ^240^Pu + ^239^Pu and Cs-137 was related to global fallout^37^. However, the timing of the ^240^Pu/^239^Pu peak was only described as sometime in the 1950s because finer resolution could not be achieved using this dataset due to bioturbation.

A site closer to the river mouth, where sites are characterized by higher sedimentation rates, was also studied for the Yangtze River estuary to identify the depth distributions of Pu-isotopes and ^137^Cs^60^. The core (140 cm in length) was retrieved from very shallow water in the estuary (ca. 15 m in water depth) and variations of Pu-isotopes were measured using AMS. The results showed that the core preserved the history of above ground nuclear testing with respect to both global fallout and PPG close-in fallout^60^. An approximately eightfold increase in both Pu-isotopes and ^137^Cs activities were seen at 70 cm bsf suggesting that the global fallout signal is recorded in this horizon. The ^240^Pu/^239^Pu ratio was around 0.2 for most of the sections, hence the majority of Pu was derived via global fallout, whereas high ^240^Pu/^239^Pu (i.e. > 0.3) are observed below 100 cm bsf suggesting that Pu isotopes derived from PPG were deposited at the East China Sea. These studies demonstrated that high resolution marine sediments obtained from the North Western Pacific using Pu-isotopes could identify the horizons of PPG close-in fallout signals during the 1950s and global fallout signals corresponding to 1963–1964 CE precisely.

The present study reports Pu-isotopes on the core obtained from Beppu Bay, Japan (Fig. 2). High resolution down core analysis of Pu-isotopes shows a clear peak of ^240^Pu/^239^Pu greater than 0.3 at 52.5 cm bsf in our master core, BMC19. The ratio drops sharply below 0.18 for depths greater than 54 cm bsf. According to the coral-based radiocarbon and uranium signatures described above, this horizon corresponds to 1955.6 CE (Fig. 4). The ^240^Pu/^239^Pu in sections shallower than 53.5 cm bsf also see a drop in their atomic ratio but maintain a ^240^Pu/^239^Pu value above 0.18. A ^240^Pu/^239^Pu higher than 0.18 is found for the upper part of the core (shallower than 54 cm bsf) suggesting a continuous contribution from the PPG through time as reported previously^37,39^. The values suggest that the particles from the PPG have contributed continuously even after the 1960s. The reproducibility of signals was confirmed by measuring twelve Plutonium samples on the core BMC 21 also obtained from Beppu Bay (Fig. 1). Remarkable resemblance of the signals was observed both for isotopic ratios and total plutonium contents in the sediment (Supplementary Figs. S1, S2). The result of ^240^Pu/^239^Pu higher than 0.18 is similar to that reported from other North Western Pacific seafloors^26^ including down core analyses in Sagami Bay, Japan^37^. Measurement of Pu in sediment obtained from Hiroshima-bay^40^ located further inside of the Seto Inland Sea (Fig. 1b) showed a similar Pu isotopic ratio throughout the core and does not exceed 0.3, which suggests that these cores did not reach horizons pertaining to 1964 CE or 1955 CE. Thus, the current study using the sediment obtained from Beppu Bay is the first record to clearly capture the key signals of the peak in activity and elevated ratios in the Pacific basin with high temporal resolution (Figs. 2 and 4).

Total ^239^Pu + ^240^Pu activities in BMC19 core reach their maximum of 7.14 mBq/g at 48.5 cm bsf and then sharply drop to ca. 2.09 mBq/g. Assigning the horizon with the highest Pu isotope ratio to 1955.6 CE and applying a linear sedimentation rate the 48.5 cm bsf horizon becomes 1964 CE when applying a linear sedimentation rate (Fig. 4). The depth is just below the event layer (Ev-1a) as is readily recognized in Beppu Bay (Fig. 2). The CT scan images of both BMC19 S1-2 and S1-3 show distinct layers that can be recognized as turbidite deposited from past geological events, such as earthquakes and tsunamis, whose ages are determined using historical documents and Pb-210 dating^1^. This particular layer (i.e. Ev-1a) was deposited as a marine turbidite resulting from the 1968 CE Hyuganada Earthquake (Mw = 7.5) off the Bungo Channel (Fig. 1b) documented in various historical records^24^. This is consistent with ^137^Cs activity measurements in the core (BMC19 S1-3) suggesting that the peak corresponding to the 1964 appeared at the horizon just below EV-1a. Thus, the timing of the total ^239^Pu + ^240^Pu activity maximum, found just below the Ev-1a (1968CE) in the sediments determined using coral radionuclides data, together with ^240^Pu/^239^Pu in the Beppu bay sediments inferred as 1964 CE, correlate well with the tight constraints provided by historical documents, and with the ^137^Cs activity peak. No major changes are detected around 2011 CE at the Fukushima Daiichi Nuclear Power Plant accident (Fig. 4) confirming the earlier findings^61^.

We propose a method for coupling coral radiocarbon and Pu isotopes signals in anoxic sediments to precisely identify the start of the Nuclear Era with potential for use as a stratigraphic marker for the Anthropocene. Coral from Ishigaki island and sediment from Beppu Bay were used to demonstrate the efficacy of this method. The nearshore sediments from anoxic seafloor environments that preserve annual layers are one of the best archives for recording changes in local and global climate relevant to anthropogenic activities. Thus, the records will be well suited to identify the baseline of natural variability, to better understand human impact on the environment, and identify the start of the Anthropocene.

## Methods

Data reported here using coral samples were previously published^13^. Briefly, the methods to produce coral-based bomb signals are described. Slabs of coral core samples were prepared for X-ray photography and micro-milling to conduct chemical analysis. Coral skeletal powders were collected along the growth axis every 0.8 mm and Sr/Ca values were measured using ICP-OES^48^. The age model was established by combining layers determined with visual counting of X-ray photographs and seasonal changes of sea surface temperature (SST) reconstructions using Sr/Ca (Fig. 3). Age uncertainties using above-described methods are approximately 1–2 months^58^.

For the measurements of radiocarbon, approximately 6–10 mg of coral powder was used. Target graphite samples were prepared using a high-vacuum line and measured using the single stage AMS located at the Atmosphere and Ocean Research Institute, The University of Tokyo^62^.

Sediment samples were carefully sliced into 1 cm intervals for various chemical analyses. The ^137^Cs content in the core was measured using a Ge-detector equipped with a multichannel analyzer at the Center for Marine Environmental Studies, Ehime University, Japan^43^.

Pu isotopes were measured using Accelerator Mass Spectrometry (AMS) at the Australian National University with an operational terminal voltage of 4 MV^63^. Owing to its high sensitivity, AMS has the advantage in measuring trace Pu isotopes using only a fraction of the core material to obtain high resolution data.

## Supplementary Information

Supplementary Information.

## Data Availability

Data presented in this paper is provided as tables in supplementary section. Also, any related materials regarding this study are available from the corresponding author upon reasonable request.
