# Supplementary material for: Plutonium isotopes in the North Western Pacific sediments coupled with radiocarbon in corals recording precise timing of the Anthropocene
Source: Sci Rep. 2022 Jul 1;12:10068. doi: 10.1038/s41598-022-14179-w (PMC9249778; doi:10.1038/s41598-022-14179-w)
Supplement: Supplementary file 1 — Supplementary Information. [file 41598_2022_14179_MOESM1_ESM.docx]

**Supplementary Information:**

**Plutonium isotopes in the North Western Pacific sediments coupled with radiocarbon in corals recording precise timing of the Anthropocene**

**Yusuke Yokoyama^1,2,3,4,5*^, Stephen Tims^5^, Michaela Froehlich^5^, Shoko Hirabayashi^1^, Takahiro Aze^1^, L. Keith Fifield^5^, Dominik Koll^5^, Yosuke Miyairi^1^, Stefan Pavetich^5^, and Michinobu Kuwae^6^**

^1^Atmosphere and Ocean Research Institute, The University of Tokyo, 5-1-5 Kashiwanoha, Kashiwa, Chiba 277-8564, Japan

^2^Department of Earth and Planetary Sciences, Graduate School of Science, The University of Tokyo, 7-3-1 Hongo, Bunkyo-ku, Tokyo 113-8033, Japan

^3^Graduate Program on Environmental Sciences, The University of Tokyo, 3-8-1 Komaba, Meguro-ku, Tokyo 153-8902, Japan

^4^Biogeochemistry Research Center, Research Institute for Marine Resources Utilization, Japan Agency for Marine-Earth Science and Technology, 2-15 Natsushima-cho, Yokosuka, Kanagawa 237-0061, Japan

^5^Research School of Physics, The Australian National University, Canberra, Acton, ACT 2601, Australia

^6^Center for Marine Environmental Studies, Ehime University, 2-5 Bunkyo-cho, Matsuyama, Ehime, 790-8577, Japan

*Corresponding author: Yusuke Yokoyama (yokoyama@aori.u-tokyo.ac.jp)

**Table S1.** Beppu Bay Pu and age depth information for BMC-19

**Table S2.** Beppu Bay Pu and age depth information for BMC-21

**Table S3.** High resolution radiocarbon data obtained from Guam and Ishigaki (1/4)

**Table S3.** High resolution radiocarbon data obtained from Guam and Ishigaki (2/4)

**Table S3.** High resolution radiocarbon data obtained from Guam and Ishigaki (3/4)

**Table S3.** High resolution radiocarbon data obtained from Guam and Ishigaki (4/4)


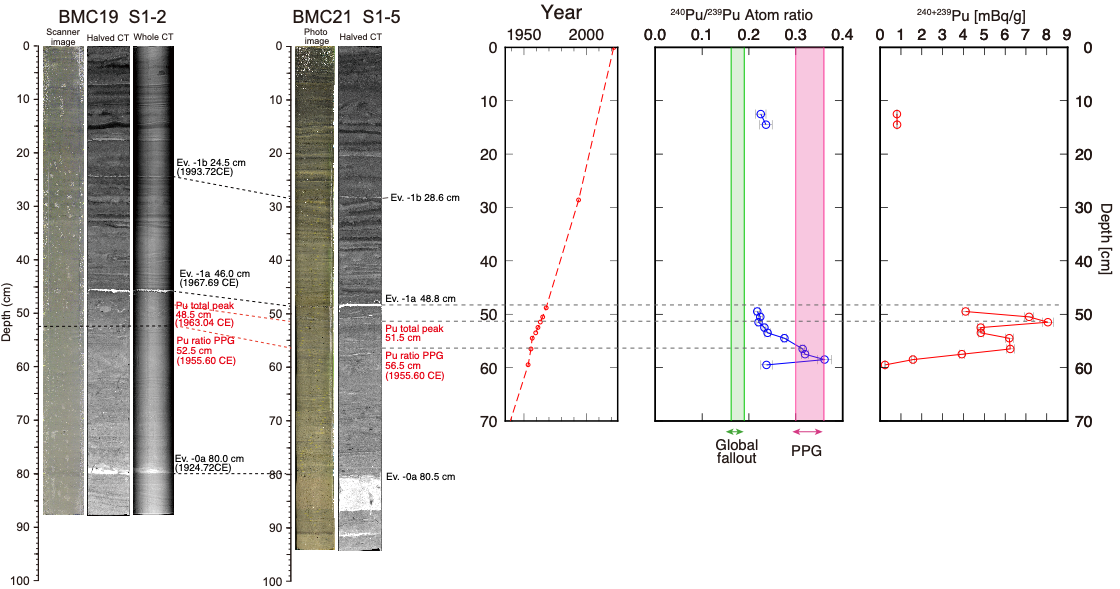


**Figure S1.** Correlation between BMC 19 S-1 and BMC-21

Well correlated layers observed in both cores in CT images can be observed. Both magnitude and shapes of Pu isotope signals in the core BMC 21 similar to the one that is observed in the master core BMC 19 (Fig. 2) are well identified.


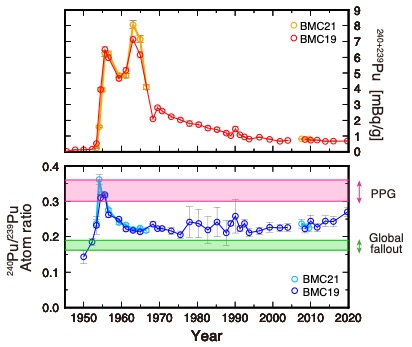


**Figure S2.** Total Pu and Pu isotopes results for BMC 19 S-1 and BMC-21

Plutonium isotope results found in both cores are almost identical.
